# The Use of Heart Rate Variability-Biofeedback (HRV-BF) as an Adjunctive Intervention in Chronic Fatigue Syndrome (CSF/ME) in Long COVID: Results of a Phase II Controlled Feasibility Trial

**DOI:** 10.3390/jcm14155363

**Published:** 2025-07-29

**Authors:** Giulia Cossu, Goce Kalcev, Diego Primavera, Stefano Lorrai, Alessandra Perra, Alessia Galetti, Roberto Demontis, Enzo Tramontano, Fabrizio Bert, Roberta Montisci, Alberto Maleci, Pedro José Fragoso Castilla, Shellsyn Giraldo Jaramillo, Peter K. Kurotschka, Nuno Barbosa Rocha, Mauro Giovanni Carta

**Affiliations:** 1 Department of Medical Sciences and Public Health, University of Cagliari, 09124 Cagliari, Italy; 2 Department of Life and Environmental Sciences, University of Cagliari, 09124 Cagliari, Italy; 3 Department of Public Health and Pediatrics Sciences, University of Torino, 10124 Torino, Italy; 4 PhD Program in Tropical Medicine, Universidad Popular del Cesar, Valledupar 200001, Colombia; 5 Microbiology Program, Universidad Popular del Cesar, Valledupar 200001, Colombia; 6 Faculty of Health, Universidad Popular del Cesar, Valledupar 200001, Colombia; 7 Department of General Practice, University Hospital Würzburg, 97080 Würzburg, Germany; 8 Center for Translational Health and Medical Biotechnology Research (TBIO), Health Research Network (RISE-Health), E2S, Polytechnic of Porto, R. Dr. António Bernardino de Almeida, 400, 4200-072 Porto, Portugal; 9 Department of Nursing, Universidad Popular del Cesar, Valledupar 200001, Colombia

**Keywords:** heart rate variability biofeedback (HRV-BF), Long COVID, chronic fatigue syndrome/myalgic encephalomyelitis (CFS/ME), feasibility study

## Abstract

**Background:** Emerging evidence indicates that some individuals recovering from COVID-19 develop persistent symptoms, including fatigue, pain, cognitive difficulties, and psychological distress, commonly known as Long COVID. These symptoms often overlap with those seen in Chronic Fatigue Syndrome/Myalgic Encephalomyelitis (CFS/ME), underscoring the need for integrative, non-pharmacological interventions. This Phase II controlled trial aimed to evaluate the feasibility and preliminary efficacy of Heart Rate Variability Biofeedback (HRV-BF) in individuals with Long COVID who meet the diagnostic criteria for CFS/ME. Specific objectives included assessing feasibility indicators (drop-out rates, side effects, participant satisfaction) and changes in fatigue, depression, anxiety, pain, and health-related quality of life. **Methods:** Participants were assigned alternately and consecutively to the HRV-BF intervention or Treatment-as-usual (TAU), in a predefined 1:1 sequence (*quasirandom assignment*). The intervention consisted of 10 HRV-BF sessions, held twice weekly over 5 weeks, with each session including a 10 min respiratory preparation and 40 min of active training. **Results:** The overall drop-out rate was low (5.56%), and participants reported a generally high level of satisfaction. Regarding side effects, the mean total Simulator Sickness Questionnaire score was 24.31 (SD = 35.42), decreasing to 12.82 (SD = 15.24) after excluding an outlier. A significantly greater improvement in severe fatigue was observed in the experimental group (H = 4.083, *p* = 0.043). When considering all outcomes collectively, a tendency toward improvement was detected in the experimental group (binomial test, *p* < 0.0001). **Conclusions:** HRV-BF appears feasible and well tolerated. Findings support the need for Phase III trials to confirm its potential in mitigating fatigue in Long COVID.

## 1. Introduction

Chronic fatigue syndrome, or myalgic encephalomyelitis (CFS/ME), is a severe, disabling condition affecting multiple organ systems, with variable symptoms depending on severity and disability level [1]. According to the US National Academy of Medicine, diagnosis requires three key symptoms: (1) a substantial reduction in pre-illness activities for more than 6 months due to unexplained fatigue unrelieved by rest; (2) post-exertional malaise (PEM), where even minimal effort triggers delayed symptom exacerbation; and (3) unrefreshing sleep. Additionally, cognitive impairment or orthostatic intolerance must be present [2]. A literature review conducted in the pre-COVID era reported an average prevalence of 1.40 ± 1.57% for this disease, with a higher incidence observed in women [3]. Currently, no pharmacological or non-pharmacological therapies have demonstrated proven efficacy for ME/CFS [4,5,6], and people affected by ME/CFS face significant barriers in accessing medical care and are often dissatisfied with health systems [7].

Thus, CFS/ME is emerging as a significant public health concern, given its substantial burden and the associated low health-related quality of life [5,8]. Around 20% of COVID-19 cases develop persistent symptoms within 2 to 4 months post-infection. A meta-analysis of 15 studies (47,910 patients) found that 80% reported at least one symptom at follow-up, with fatigue (58%) being the most common, followed by various types of pain such as headache, joint pain, chest discomfort, and dyspnea [9]. Some cases of Long COVID meet the diagnostic criteria for chronic fatigue syndrome/myalgic encephalomyelitis (CFS/ME). In fact, even more than 6 months after the first clinical manifestations of the infection, 13 to 23% of people hospitalized with SARS-CoV-2 infection present chronic fatigue (“PEM-like symptoms”) [10].

This clinical and epidemiological context informs the objective of the present study, which is to assess the feasibility and preliminary efficacy of Heart Rate Variability Biofeedback (HRV-BF) in individuals affected by Long COVID who meet diagnostic criteria for CFS/ME. Specifically, we hypothesized that HRV-BF would represent a feasible and acceptable intervention, potentially leading to improvements in fatigue, anxiety, depressive symptoms, pain, and health-related quality of life. This hypothesis is further supported by recent evidence indicating a substantial psychological burden among Long COVID patients. In particular, a recent systematic review and meta-analysis has demonstrated a significant prevalence of mental health issues in this population, especially symptoms of depression, anxiety, and a notable decline in quality of life [11].

Moreover, the main risk factors for persistent fatigue in Long COVID patients include advanced age, female sex, a history of depression or anxiety, and a high body mass index (BMI) [12]. Additionally, specific biomarkers such as interleukin 1 receptor, type II (IL-1R2), matrilin 2 (MATN2), and collectin-12 (COLEC12) have been associated with prolonged fatigue symptoms [13]. Notably, chronic fatigue syndrome development appears independent of initial disease severity [14]. The association with mental health conditions has led to hypotheses of a “functional” etiology, contributing to a discriminatory perception of the syndrome, amplifying what has already occurred with CFS/ME without Long COVID. Contrary to psychosomatic interpretations, over one-third of patients with post-COVID fatigue exhibit persistently elevated D-dimer levels, and 10% have increased C-reactive protein levels, suggesting an ongoing inflammatory process [15]. COVID-19 has been shown to cause activation of central nervous system (CNS) immune cells at the pathogenetic level, which may be one of the causes of the frequent chronic fatigue syndrome associated with the viral infection [16,17]. Historical data from previous coronavirus outbreaks indicate long-term neurological involvement, as observed in a cohort of 300 post-SARS patients who exhibited nervous system damage 4 years post-infection [18]. The COVID-19 pandemic may exacerbate the public health issue of CFS/ME, highlighting the urgent need for effective diagnostic and therapeutic approaches. Our research group recently conducted a Phase II trial on heart rate variability biofeedback (HRV-BF) in fibromyalgia, aiming not to assess its specific efficacy but to evaluate its ability to interrupt the anxiety–alarm cycle triggered by pain, which exacerbates the syndrome’s impact. The study confirmed the feasibility of HRV-BF, though efficacy results remained inconclusive [19], as expected for a Phase II trial [19]. A secondary analysis suggested potential improvements in fatigue-related symptoms, relevant given the overlap between fibromyalgia and Long COVID symptoms [20].

Based on these considerations, we conducted a Phase II controlled trial to assess the feasibility of Heart Rate Variability Biofeedback (HRV-BF) in individuals with Long COVID. The study aimed to evaluate dropout rates, side effects, and participant satisfaction, as well as to explore preliminary improvements in fatigue, pain perception, quality of life, depressive symptoms, and anxiety before and after the intervention.

## 2. Materials and Methods

### 2.1. Study Design

This controlled clinical feasibility trial, registered under protocol code ClinicalTrials.gov ID: NCT05793736, involved three evaluations: T0 (pre-treatment), T1 (post-treatment, 5 weeks later), and T2 (9-week follow-up). The experimental group underwent a BF-HRV training protocol (twice weekly for 5 weeks) alongside treatment as usual (TAU), while the control group continued with TAU alone. Treatment as usual consisted of analgesics and antidepressants.

### 2.2. Participants

A total of 18 participants were recruited from the Pain Unit and the Psychiatric Liaison Unit of the University Hospital of Cagliari, Italy. Recruitment occurred from February to May 2023. Inclusion criteria were: age over 18 years, both sexes, a history of acute respiratory syndrome due to SARS-CoV-2 confirmed by a positive molecular Polymerase Chain Reaction (PCR) test and fever within the past 12 months, and fulfillment of the diagnostic criteria for ME/CFS as defined by the 2015 Report from the Committee on the Diagnostic Criteria for Myalgic Encephalomyelitis/Chronic Fatigue Syndrome. Exclusion criteria included moderate to severe intellectual disability and severe comorbidities, except for anxiety or depressive symptoms emerging in association with the viral infection or Long COVID syndrome, given the high prevalence of such symptomatology in Long COVID [9].

Following written informed consent to participate, participants were assigned alternately and consecutively to the intervention or control group, in a predefined 1:1 sequence (*quasirandom assignment*). This method ensured balance between groups, Outcome assessment was blinded to group allocation. As no prior studies have employed Heart Rate Variability Biofeedback (HRV-BF) in individuals with Long COVID who meet diagnostic criteria for CFS/ME, it was not possible to base a sample size calculation on previously established effect sizes. In the absence of such data, this Phase II study aimed primarily to assess feasibility and acceptability, while providing preliminary effect estimates that may guide future adequately powered trials. Despite the limited sample, the study allows for a broader exploration of feasibility outcomes (e.g., adherence, tolerability, satisfaction), which can be inferred with greater confidence than efficacy endpoints in such early-phase trials.

### 2.3. Intervention Protocol

Biofeedback, through the use of visual or auditory signals, facilitates the enhancement of self-regulatory capacities and strengthens parasympathetic nervous system activity, thereby contributing to improved stress management and emotional regulation [21]. The physiological signals commonly monitored in biofeedback include respiration, skin temperature, blood pressure, electrodermal activity, surface electromyography [22] and heart rate variability (HRV), which is now considered a crucial marker of psychological well-being [23]. Heart Rate Variability Biofeedback (HRV-BF), a cardiorespiratory-based intervention, is widely recognized for its efficacy in promoting mental health [24], managing pain conditions such as headaches and migraines [25] and addressing sleep disorders, chronic pain syndromes [26,27], and chronic fatigue [19,28].

The HRV-BF protocol was carried out in accordance with the literature and with our experience in fibromyalgia [18,19,20]. The 10 sessions (twice weekly for 5 weeks) lasted about 50 min each. HRV data were recorded using sensors placed in the earlobe of the participant. The session was delivered by emWave equipment (HeartMath^®^, 2014, manufactured by Quantum Intech, Inc., Boulder Creek, CA, USA). Participants received real-time visual feedback on their heart rate activity, enabling them to synchronize their heart rhythms with controlled breathing patterns. Through the continuous feedback provided during the respiratory training, participants progressively learned to modulate their heart rate variability (HRV), which fostered a greater sense of relaxation, bodily awareness, and a pleasant feeling of presence. Each session consisted of an initial preparatory phase of approximately 10 min focused on respiratory regulation, followed by an active training phase lasting around 40 min. The active participation of a supervisor technician (health-trained health professional) exclusively supported the execution of breathing tasks. This support gradually decreased, promoting the person’s autonomy, aiming to enable the participant’s ability to self-induce relaxation, and to generalize it in other frameworks of their daily life, even in stress or in danger with pain. The staff involved in the project were health professionals (psychologists, psychiatrists, health educators) qualified in mental health and trained in HRV technology. Researchers strictly adhered to the protocol previously developed. Participants were clearly informed that HRV-BF was not intended to ”treat” the condition in the strict medical sense, but rather to provide a tool for managing the disorder by interrupting the short circuit of excessive alarm in response to potentially uncomfortable bodily sensations. In other words, the persistent experience of fatigue and pain can trigger a state of hyper-alertness, leading to an increased respiratory rate as soon as the individual anticipates exposure to a potential stimulus. Paradoxically, instead of serving a protective function, this response intensifies muscular tension and, as a result, exacerbates fatigue and increases vulnerability to pain. While awareness of this dysfunctional loop does not cure the condition, it can play a significant role in its management.

### 2.4. Instruments

A specific form was used for the collection of the sociodemographic variables, such as sex, age, education level, and occupation status.

### 2.5. Feasibility

Measured by the dropout rate, which is considered acceptable when below 20%, and typically around 30% in populations with chronic pain and fatigue values that are in line with those reported in the literature for randomized controlled trials (RCTs) targeting pain and fatigue management [29,30].

### 2.6. Participant Satisfaction

Participant satisfaction was assessed using a modified version of a previously validated ad hoc questionnaire [31,32]. Two additional items were included to evaluate the perceived usefulness and frequency of use of skills acquired during biofeedback training, enhancing the assessment’s ecological validity by focusing on real-life applicability. The final questionnaire comprised eight items rated on a 5-point Likert scale, covering overall judgement, effects on general health, operator and organizational support, expectations, willingness to recommend the intervention, and practical application of skills. Mean and standard deviation (SD) scores were calculated, with possible total scores ranging from 8 to 40.

### 2.7. Side Effects

Although HRV-BF is generally considered to be free of side effects, we nevertheless aimed to verify this within our sample using the Simulator Sickness Questionnaire (SSQ), developed by Kennedy et al. (1993) [33] and further analyzed by Balk (2003) [34]. The SSQ, widely used to assess side effects from virtual environments and simulators, includes 16 symptoms rated on a 4-point Likert scale (0 = None to 3 = Severe), generating a total and three subscale scores: Nausea (N), Oculomotor (O), and Disorientation (D). It was chosen for HRV-BF training due to its sensitivity in detecting subtle adverse effects, despite the less immersive nature of HRV-BF compared to VR. The intervention involved audiovisual equipment (computer, Dolby Surround 5.1 speakers, and a 50 inch display). Mean and standard deviation values for the SSQ total and subscale scores were calculated, with scoring interpreted according to the original validation thresholds [33]. Originally developed for military flight simulators, the SSQ’s application has evolved with technological advancements and clinical populations. A meta-analysis of 55 VR interventions [35] reported total SSQ mean scores ranging from 14.30 to 35.27, with an overall mean of 28.00 (95% CI: 24.66–31.35). Disorientation had the highest pooled subscale score (M = 23.50), followed by oculomotor symptoms (M = 17.09) and nausea (M = 16.72). According to Bateman et al. [36], total scores of 10–15 indicate significant symptoms, 15–20 raise concerns, and scores above 20 suggest a problematic experience. These thresholds, initially based on flight simulators [36,37], are generally higher in studies involving VR and digital media. Notably, even the lowest SSQ mean score of 14.30 reported in VR interventions with older adults [35] would indicate significant symptoms under these criteria [36].

### 2.8. Outcomes

Fatigue symptoms were measured by means of the *Brief Fatigue Inventory*, a tool originally developed in oncology [38], then used in other clinical fields [39,40] including the measure of the fatigue syndrome in Long COVID [41]. It consists of nine items, with answers coded 0 to 10. Three items enquire the severity of fatigue, with “0” corresponding with “no fatigue” and “10” corresponding with “fatigue as bad as you can imagine”. The additional items measure the interference of fatigue in aspects of daily activities; in this case, the scale ranges from “0” (no interference) to “10” (the fatigue completely interferes). The higher the score achieved, the more severe is the fatigue. Considering a general cut-off of >7 for the tool, which identifies severe fatigue, the primary outcome is defined as the number of people who improve from a severe fatigue level during the trial or, conversely, worsen to a state of severe fatigue.

Depressive symptoms were measured through *the Patient Health Questionnaire (PHQ-9)* [42], a nine-item tool conceived for screening depressive episodes and assessing and monitoring the severity of depressive symptoms, including all items concerning the Diagnostic and Statistical Manual of Mental Disorders (DSM) criteria for a diagnosis of major depressive episode, integrated into a simple self-report instrument. The tool was already used to measure depressive symptoms during the COVID-19 era [43]. The internal consistency is Cronbach’s α = 0.89 [44]. Considering the cut-off of >10 for identifying mild to severe depressive episodes [45,46], the secondary outcome is defined as the number of people who improve from mild to severe depressive episodes during the trial or, conversely, worsen from mild to severe depressive episodes. In our neo-Kraepelinian view, the use of the PHQ-9 measures the severity of the entire spectrum of mood disorders, including major depressive disorder, bipolar disorder, and stress-related disorders such as Post-Traumatic Stress Disorder (PTSD) [47,48], the association of which with CFS/ME has recently emerged [49,50].

*The Zung Self-Rating Anxiety Scale (SAS*) is a psychological assessment tool [51] conceived to measure anxiety severity. It consists of 20 self-administered items, each rated on a 4-point scale from “none or a little of the time” to “most or all of the time”. The scale evaluates cognitive, affective, somatic, and autonomic anxiety symptoms, providing a comprehensive assessment of an individual’s anxiety level. The total score reflects anxiety severity, with higher scores indicating greater symptom intensity. Widely used in clinical and research settings, the SAS allows for independent administration and is effective for tracking symptom progression and assessing treatment outcomes over time. Considering the cut-off of >50 for identifying a clinically relevant anxiety state [52,53], the secondary outcome is defined as the number of people who improve from an anxiety state during the trial or, conversely, worsen to an anxiety state.

*The Short-Form Health Survey (SF-12)* [54] is a tool designed to evaluate both the physical and psychological components of the perception of health-related quality of life (H-QoL). It consists of a 12-item questionnaire aiming to assess how health is perceived to influence an individual’s daily life. Its internal consistency is Cronbach’s α = 0.94 [55].

However, given the clinical population under investigation and in line with previous studies on chronic illnesses and quality of life in Italy, a cut-off score of 30 was adopted to identify individuals in the sample with severely low health-related quality of life [55]. Accordingly, the secondary outcome in this study is defined as the number of participants who show improvement from a state of severely low H-QoL during the trial, or conversely, those who deteriorate to a severely low H-QoL level.

*The Pain Visual Analogue Scale (PVAS)* is one of the best-known unidimensional outcomes for measuring pain intensity. It represents a visual depiction of the extent of pain experienced by the patient and consists of a predefined 10 cm-long line, where the left end corresponds to “no pain” and the right end to “worst possible pain [56,57,58]. The patient is asked to mark a point on the line that best represents their pain level. The Visual Analogue Scale (VAS) is considered an ordinal one [59]. For its administration, it requires a minimum level of visuomotor coordination [41]. Test–retest reliability was found to be high, with a Pearson correlation from r = 0.94 to r = 0.71 in illiterate patients [60,61]. Considering the cut-off of >45, for mild to severe pain, based on a scoring scale from 0 to 100 (0 = no pain, 100 = worst possible pain), [56,57] the secondary outcome is defined as the number of people who improve from mild to severe pain during the trial or, conversely, worsen to mild from severe pain.

### 2.9. Statistical Analysis

In the statistical analyses we had to take into account (1) that these were analyses of a preliminary two-phase study on samples of small size, (2) that sometimes the assumption of the normal distribution of the populations from which the samples were supposed to be extracted was not respected, and (3) that some instruments such as visual pain were declared to be on ordinal measures and not on numerical intervals. The statistical analyses of comparison between groups were therefore conducted on ordinal scales by establishing at T1 three levels for each group and for each measure; that is, the first level was the number of individuals with an improvement in outcome from T0 to T1, the second level was the number of individuals with an unchanged outcome level from T0 to T1, and the third level was the number of individuals with the outcome under examination worsened from T0 to T1.

This categorical approach was chosen due to the small sample size and the non-normal distribution of several variables, which limited the use of parametric methods. Furthermore, defining discrete levels of change allowed for a more interpretable and clinically meaningful analysis of individual symptom trajectories in a feasibility framework.

Comparison for each outcome measure (one primary and four secondary) was conducted using the Kruskal–Wallis test. Measurement of the homogeneity of the trends in the five measurements was conducted using a binomial test. Verification of the acceptance of the hypothesis that the hypothetical reference populations of the samples had a normal distribution was conducted with the Shapiro–Wilks test.

There were no missing data at the level of outcome measures; thus, no imputation techniques were required.

### 2.10. Ethical Considerations

The study protocol has been approved by the Independent Ethics committee, University hospital of Cagliari (reference number: NP/2023/496). All included subjects provided written informed consent. All human procedures followed were in accordance with the guidelines of the Helsinki Declaration of 1975 [62].

## 3. Results

Table 1 summarizes the sociodemographic characteristics and baseline outcome measures of the two groups. Overall, the experimental and control samples were comparable in age, education level, baseline fatigue, depression, anxiety, quality of life, and pain (all *p* > 0.05). In particular, there was also a balance between the experimental and control groups regarding the presence of more pronounced depressive symptoms at screening. While the control group consisted entirely of women, sex distribution did not differ significantly (*p* = 0.165). No statistically significant differences were observed between groups on any outcome at T0.

### 3.1. Feasibility (Drop-Out Rate)

Only one participant—a 51-year-old woman—discontinued participation in the experimental group due to serious health issues unrelated to the study procedures. The overall drop-out rate was 5.56% (1 out of 18 participants). In the experimental group, one participant (11.1%) discontinued participation due to reasons unrelated to the study, while no drop-outs occurred in the control group (0%).

### 3.2. Participant Satisfaction

The total mean score obtained from the questionnaire was 34.37 (SD = 3.2), within a possible range from 8 to 40, indicating a generally high level of satisfaction among participants.

### 3.3. Side Effects

To quantitatively characterize the distribution of simulator-induced symptoms, we calculated descriptive statistics (mean and standard deviation) for the total score and the three subscales—Nausea (N), Oculomotor (O), and Disorientation (D)—of the Simulator Sickness Questionnaire (SSQ). The results showed a total score with a mean of 24.31 and a standard deviation (SD) of 35.42. For the Nausea subscale, the mean was 16.95 (SD = 32.55); for the Oculomotor subscale, the mean was 20.84 (SD = 23.88); and for the Disorientation subscale, the mean was 27.84 (SD = 43.38). It is important to note that within the experimental sample (N = 8), one participant was identified as an outlier, presenting substantially higher scores than the rest of the group, with a total score of 104.72; Nausea subscale = 95.4; Oculomotor subscale = 68.22; and Disorientation subscale = 125.28. Therefore, we also report descriptive statistics with the outlier excluded, which yielded a total score mean of 12.82 (SD = 15.24), a Nausea subscale mean of 5.45 (SD = 7.5), an Oculomotor subscale mean of 14.07 (SD = 15.42), and a Disorientation subscale mean of 13.92 (SD = 19.68).

### 3.4. Outcome Measures

The frequency of people with severe fatigue was balanced in the two samples at the beginning of the trial, i.e., four (50%) in the experimental group versus two (22.22%) in the control group (Fisher Exact Test, *p* = 0.335), as well as the distribution of the severity level of the fatigue syndrome measured by the BFI scale score (6.44 ± 1.94 vs. 6.36 ± 1.29; H = 0.070, *p* = 0.791, Kruskal–Wallis Test). Similarly, the other outcome measures examined were also balanced: four (50%) people with mild or more severe depression in the experimental group versus six (66.67%) in the control group (Fisher Exact test, *p* = 0.637); six (75%) people with a state of anxiety in the experimental group versus eight (88.89%) in the control group (Fisher Exact test, *p* = 0.559); five (62.5%) people with very low levels of H-QoL in the experimental group versus six (77.78%) in the control group (Fisher Exact test, *p* = 0.929); four people (50%) with moderate to severe pain in the experimental group versus four (44.44%) in the control group (Fisher Exact test, *p* = 0.929). As shown in Table 1, the scores on the respective scales (PHQ-9, SAS, SF-12 and PVSAS) for the other outcome measures also did not show statistically significant differences. The possibility of hypothesizing a normal distribution of the scores of the examined items is not confirmed by the Shapiro–Wilk Test in the SAS scores of the control sample (*p* = 0.043).

The difference in outcome in the experimental and control groups is illustrated in Table 2. In the experimental group, a better effect is observed with regard to severe fatigue, that is, three of the people with a severe level of fatigue at the beginning of the trial no longer have such a severe level at T1, compared to no improvement in the control group. On the other hand, two people who did not have such a severe level at T0 worsened in the control group, compared to no worsening in the experimental group (H = 4.083—*p* = 0.043, Kruskal–Wallis Test).

A similar improvement is not observed for depression (H = 0.079, *p* = 0.701, Kruskal–Wallis test); for those individuals who struggle with anxiety (H = 0.194, *p* = 0.658, Kruskal–Wallis test); people with low quality of life (H = 0.007, *p* = 0.929, Kruskal–Wallis test); and for people with medium to severe pain (H = 0.175, *p* = 0.0.894, Kruskal–Wallis test).

Although individual outcomes did not reach statistical significance, a consistent pattern of more favorable changes was descriptively observed in the experimental group. This sequence was statistically significant according to the binomial test (*p* < 0.0001); however, given the exploratory nature of the trial and small sample size, this result should be interpreted with caution.

When aggregating changes across the five outcome domains, a higher proportion of improvements was observed in the experimental group (27.5%) compared to the control group (6.67%), with no cases of worsening in the experimental group versus 6.67% in the control group. While this difference reached statistical significance (H = 4.136, *p* = 0.041 Kruskal–Wallis Test), it should be interpreted as an exploratory signal rather than definitive evidence of treatment effect.

## 4. Discussion

This feasibility phase II study yields some interesting main results. First, the trial shows a low dropout rate (11%) and a high level of satisfaction for the intervention was found among participants. About the side effects, the mean total SSQ score and subscale scores appear broadly consistent with the symptom profiles reported in more recent VR research [35]. This suggests that they are not unusually elevated compared to those found in other digital media exposure studies. Furthermore, it is important to note that these averages, although in line with previous studies, were substantially influenced by a single participant who reported extremely high symptom scores across all SSQ dimensions. When this outlier was excluded, the overall symptom profile changed significantly and fell below the typical ranges reported in VR-related studies and below Kennedy’s particularly restrictive threshold of 15 which denotes the presence of “significant” symptoms. This suggests that the technique may be even better tolerated and practically free of side effects, as is typically observed in the use of biofeedback. Moreover, this case highlights that some individual variability in response to the technique is to be expected. However, extreme deviations from the mean such as this should be interpreted with caution and more accurately characterized in larger samples. Considering these outcomes as a whole, it can reasonably be stated that the intervention demonstrated a good level of feasibility. Preliminary data regarding the improvement induced by the HRV-BF technique on the outcomes of interest suggest that the intervention may hold promise, pending confirmation in future studies. Notably, a 37.5% greater proportion of participants improved in fatigue symptoms compared to the control group should be interpreted with caution, given the inherent limitations of a Phase II study, the small sample size, and the associated risk of a Type I error.

Regarding dropout rates, an essential aspect of acceptance and feasibility in studies, especially within this clinical population, it should be emphasized that the result appears very encouraging. A Cochrane systematic review [63] on randomized controlled trials employing cognitive behavioral therapy (CBT) for CFS/ME highlighted the critical challenges faced by non-pharmacological interventions. The average dropout rate in CBT studies was slightly above 15%, ranging from 0% [64] to 40% [65], with many studies reporting rates exceeding 20% [65,66,67,68,69,70]. More recent studies confirm dropout rates above 20% [71]. In trials comparing CBT with other active psychological therapies or graded exercise therapy, only Jason’s study [70] found no significant differences across intervention groups, with dropout rates around 25%. Other non-pharmacological interventions reported comparable or even higher dropout rates: 13.3% for relaxation therapy [72], 31.3% for counseling [67], and 28.6% to 40% for graded exercise therapy [69]. Slightly lower rates were observed in education or psychoeducation control groups, ranging from 0% in an unpublished survey [73], to 30.9% for guided support [66] and 10% for education and support [74]. Additionally, a more recent study not included in the Cochrane review investigated a group-based lifestyle education program and reported a 3-month dropout rate of 15.8% [75].

However, given its exploratory nature and the small sample size typical of Phase II studies, the findings should be considered only as preliminary and suggestive of further investigations, particularly in relation to treatment efficacy. It is possible that the intervention’s structure—specifically, the emphasis on providing a management tool rather than directly treating the disorder, the active involvement of participants, and the structured relaxation phases—played a significant role in influencing retention rates.

The review of CBT shows (on four studies about 371 participants) a clinical response in the short term of 40% for CBT compared to 26% for usual care, so an advantage of just over 20% [63]. However, the interpretation of the efficacy studies of CBT is not univocal [4], and the associations of patients with CFS/ME express strong doubts about its use [76]. CBT may have even less effect if there is comorbidity with depression [77]. If this were confirmed, it would represent a serious problem because comorbidity with depression is very common, especially in the most severe forms [77]. In contrast, as HVB-BF could influence the biorhythm system [78,79,80], this aspect could be of particular interest in respect to stress, anxiety, and mood syndromes [81]; thus, in the frequent case of co-morbidity between these disorders and chronic fatigue syndromes. Compared to CBT, the HVB-BF intervention does not claim to be therapeutic, at least in the sense that it does not aim to cure but simply to provide a tool to help the person in managing the disorder. Furthermore, it entails a more autonomous and immediate role for the user, both in applying the technique during training sessions and in independently implementing it in real-life stressful situations. For this reason, it might be that it is better accepted, and this can explain the very low dropout rate achieved. Another systematic review, recently updated, conducted specifically on exercise therapy showed a possible (moderate) effect but without evident advantages compared to CBT or antidepressants [82].

The use of exercise has itself been the subject of fierce controversy. Since the publication of the previous NICE guidelines (2007) [4] and the PACE Trial (2011), the ME Association has been vehement in its argument that the use of graded, regulated and inflexible exercise therapy as a management approach for people with ME/CFS is wrong, ineffective, and can cause harm. The ME Association has called for NICE to withdraw its recommendation for the use of exercise, pointing out that research evidence has found methodological weaknesses in clinical trials proving the effectiveness of exercise and that surveys of the opinions of people who have used this therapy have shown that this treatment method can be perceived as ineffective and harmful [76]. NICE then completely revised the ME/CFS guideline and, after a full assessment of the evidence [83], withdrew its support for the use of exercise in CFS/ME. The new NICE guidelines warn against the use of exercise in Long COVID [4]. The case of Paul Garner, a professor in Liverpool, is emblematic of these controversies [84]. Garner was affected by severe CFS and Long COVID. After participating in an intensive physical exercise program, he relapsed dramatically with severe fatigue syndrome [84]. After the use of techniques to reduce symptoms and related stress, Garner slowly recovered. His journey led him to the belief that “post-exertional” malaise after an exercise plays a role in people with CFS/ME, as an automatic learnt brain response [84]. The ME patient associations, which were always against a “psychologizing” interpretation of the syndrome, turned against Garner. The purpose of a typical physical exercise training was thus considered a “psychologizing” interpretation [84]. It may be somewhat ambiguous because today physical exercise is known to produce bio-physiological consequences [85,86]. But this is understandable because in a recent survey of over 2000 people suffering from ME/CFS, most reported worsening of symptoms after physical exercise [87]. Garner himself reiterated that, while there is still a tendency to “psychologize” a problem for which there are no clear solutions, one must also recognize that excessive medicalization of the issue, reinforced by media coverage, fosters the belief in irreversible biological damage and, consequently, amplifies the impact [88]. This impasse could be addressed by adopting approaches that enable healthcare professionals to offer supportive interventions which, while not claiming to be curative, may enhance patients’ ability to manage their condition. If confirmed to yield these preliminary improvements, biofeedback could play a meaningful role in this regard. Firstly, because the role of the person suffering from the disorder can have a much more autonomous and independent role than in CBT, where, by definition, the role of the therapist is pivotal, and secondly, because the intervention could present fewer side effects than physical exercise, could be more accessible and, ultimately, would marry very well with other therapies aimed at the “physiopathogenetic” treatment of the disorder.

### Strengths and Limitations

Given the exploratory nature and limited sample size of this Phase II trial, the present study was not powered to evaluate the clinical efficacy of HRV-BF, but rather to assess its feasibility and acceptability, and to explore preliminary outcome trends. Importantly, no prior studies have employed HRV-BF in individuals with Long COVID who meet diagnostic criteria for CFS/ME, precluding formal a priori power calculations. In this context, the inclusion of multiple outcome measures—while potentially increasing the risk of a type I error—was intended to capture a broad range of symptom dimensions relevant to the population and intervention under study. Nevertheless, we recognize that the use of several psychometric scales in a small sample may dilute focus and lead to overinterpretation of weak or non-robust effects. For this reason, the interpretation of changes based on clinical cut-offs (e.g., improvement vs. worsening) should be considered purely exploratory and descriptive, not inferential. On the other hand, one of the key strengths of this study is the assessment of feasibility indicators, including dropout rates, adverse events, and perceived utility of biofeedback techniques. The addition of items measuring the frequency and context of technique use adds ecological validity, suggesting that participants may be able to integrate self-regulatory skills into daily life routines. Future studies should further explore these aspects, ideally incorporating qualitative methodologies (e.g., semi-structured interviews) to better understand how individuals implement and benefit from HRV-BF in real-world settings.

## 5. Conclusions

In addition to confirming feasibility and tolerability, future Phase III studies are needed to validate the potential role of HRV-BF in attenuating fatigue symptoms in CFS/ME associated with Long COVID, as suggested by these preliminary findings. Future trials should also have the power to investigate whether those components evaluated for which the present work has not shown reliable evidence can also benefit from the intervention. In the evidence of a frequently reported association of CFS with mood spectrum disorders and stress syndromes, it should also be verified whether in this field biofeedback can also be used as a tool for regulating biological and social rhythms.

## Figures and Tables

**Table 1:** Sociodemographic characteristics and outcome measures of the sample at T0.

	Experimental Group (N = 8)	Control Group (N = 9)		
**Age**	49.75 ± 9.98	49.33 ± 19.33	H = 0.037 Kruskal–Wallis Test	*p* = 0.837
**Sex (female)**	5 (62.5%)	9 (100%)	Fisher Exact Test	*p* = 0.165
**Graduated or** **More**	3 (37.8%)	5 (55.5%)	Fisher Exact Test	*p* = 0.637
**Brief Fatigue Inventory score**	6.44 ± 1.94	6.36 ± 1.29	H = 0.070 Kruskal–Wallis Test	*p* = 0.791
Shapiro–Wilk Test	*p* = 0.850	*p* = 0.430		
**With severe Fatigue**	4 (50%)	2 (22.22%)	Fisher Exact Test	*p* = 0.335
**PHQ-9 score**	6.44 ± 1.94	6.36 ± 1.29	H = 0.079 Kruskal–Wallis Test	*p* = 0.701
Shapiro–Wilk Test	*p* = 0.850	*p* = 0.430		
**With depression mild or more**	4 (50%)	6 (66.67%)	Fisher Exact Test	*p* = 0.637
**Self-Rating Anxiety Scale Score**	56.66 ± 11.01	57.00 ± 8.51	H = 0.194 Kruskal–Wallis Test	*p* = 0.658
Shapiro-Wilk Test	*p* = 0.931	*p* = 0.049		
**People with Anxiety State**	6 (75%)	8 (88.89%)	Fisher Exact Test	*p* = 0.620
**SF-12 score**	23.77 ± 3.57	24.00 ± 3.552	H = 0.007 Kruskal–Wallis Test	*p* = 0.929
Shapiro–Wilk Test	0.490	0.790		
**People with low H-QoL**	5 (62.5%)	7 (77.78%)	Fisher Exact Test	*p* = 0.999
**Pain Visual Analogue score**	41.05 ± 27.30	44.00 ± 32.06	H = 0.175 Kruskal–Wallis Test	*p* = 0.894
Shapiro–Wilk Test	0.999	0.669		
**People with** **moderate pain or more**	4 (50%)	4 (44.44%)	Fisher Exact Test	*p* = 0.999

**Table 2:** The difference in outcomes measures in experimental and control groups after intervention.

	Remission from Condition	Not Changed	Condition Appeared to Worsen	
**Severe Fatigue**				
Experimental group	3 (37.5%)	5 (62.5%)	0 (0%)	Kruskal–Wallis test
Control group	0 (0%)	7 (77.78%)	2 (13.33%)	H = 4.083—*p* = 0.043
**Depression mild or more**				
Experimental group	2 (22.2%)	6 (77.8%)	0 (0%)	Kruskal–Wallis test
Control group	0 (0%)	8 (88.9%)	1 (11.9%)	H = 0.079, *p* = 0.701
**Anxiety State**				
Experimental group	2 (25%)	6 (75%)	0 (0%)	Kruskal–Wallis test
Control group	1 (11.11%)	8 (88.89%)	0 (0%)	H = 0.231—*p* = 0.630
**Low H-QoL**				
Experimental group	3 (37.5%)	5 (62.5%)	0 (0%)	Kruskal–Wallis test
Control group	1 (11.11%)	8 (88.89%)	0 (0%)	H = 0.836—*p* = 0.360
**Moderate pain or more**				
Experimental group	1 (12.50%)	7 (87.5%)	0 (0%)	Kruskal–Wallis test
Control group	0 (0%)	9 (100%)	0 (0%)	H = 0.187—*p* = 0.669
**Total improvements**				
Experimental group	11 (27.5%)	29 (72.5%)	0 (0%)	Kruskal-Wallis test
Control group	3 (6.67%)	39 (86.66%)	3 (6.67%)	H = 4.136—*p* = 0.041

## Data Availability

The datasets of this study will not be publicly available due to individual privacy rules.
